# Low Adoption of Video Consultations in Post–COVID-19 General Practice in Northern Europe: Barriers to Use and Potential Action Points

**DOI:** 10.2196/47173

**Published:** 2023-05-22

**Authors:** Elisabeth Assing Hvidt, Helen Atherton, Jelle Keuper, Eli Kristiansen, Elle Christine Lüchau, Børge Lønnebakke Norberg, Jost Steinhäuser, Johannes van den Heuvel, Lilian van Tuyl

**Affiliations:** 1 Research Unit of General Practice Department of Public Health University of Southern Denmark Odense Denmark; 2 Unit of Academic Primary Care Warwick Medical School Coventry United Kingdom; 3 Netherlands Institute for Health Services Research Utrecht Netherlands; 4 Tranzo Tilburg School of Social and Behavioral Sciences Tilburg Universit Tilburg Netherlands; 5 Norwegian Centre for E-health Research University Hospital of North Norway Tromsø Norway; 6 Institute of Family Medicine University Medical Center Schleswig-Holstein, Campus Luebeck Luebeck Germany; 7 Center for General Practice Aalborg University Aalborg Denmark

**Keywords:** video consultation, adoption, general practice, Northern Europe, barriers, action potential, Europe, viewpoint, consultation, barrier, clinician, digital care, care, implementation, practitioner, COVID-19, research

## Abstract

In the wake of the COVID-19 pandemic, video consultation was introduced in general practice in many countries around the world as a solution to provide remote health care to patients. It was assumed that video consultation would find widespread adoption in post–COVID-19 general practice. However, adoption rates remain low across countries in Northern Europe, suggesting that barriers to its use exist among general practitioners and other practice staff. In this viewpoint, we take a comparative approach, reflecting on similarities and differences in implementation conditions of video consultations in 5 Northern European countries’ general practice settings that might have created barriers to its use within general practice. We convened at a cross-disciplinary seminar in May 2022 with researchers and clinicians from 5 Northern European countries with expertise in digital care in general practice, and this viewpoint emerged out of dialogues from that seminar. We have reflected on barriers across general practice settings in our countries, such as lacking technological and financial support for general practitioners, that we feel are critical for adoption of video consultation in the coming years. Furthermore, there is a need to further investigate the contribution of cultural elements, such as professional norms and values, to adoption. This viewpoint may inform policy work to ensure that a sustainable level of video consultation use can be reached in the future, one that reflects the reality of general practice settings rather than policy optimism.

## Introduction

Video consultation—defined here as a synchronous communication between the general practitioner (GP) and the patient using video calling [1]—was barely in use in general practice in countries in Northern Europe before the COVID-19 pandemic (Table 1).

During the COVID-19 pandemic, when video consultation was abruptly introduced in general practice, there was a strong belief that the pandemic would serve as “the window of opportunity” [2] for its widespread and long-term adoption. However, although levels of use of video consultation increased during the acute phase of the COVID-19 pandemic, larger scale deployment of video consultations in post–COVID-19 general practice in Northern Europe is yet to be seen [1,3-7].

Studies on patient experiences with video consultation in general practice show that patients tend to show high satisfaction, valuing aspects such as convenience, flexibility, and efficiency [8-11]. It should be noted, however, that a major factor influencing patient satisfaction levels and desire for more digital care in general practice in the future is appropriateness and suitability of use [8,11,12]. A study using a discrete choice experiment, which investigated attributes influencing patients’ hypothetical choice between face-to-face in-clinic consultation and telemedicine consultations (eg, video, telephone, and text-based consultations), showed that patients preferred video consultation over face-to-face consultation if it meant that the waiting time until an appointment and queuing time were shorter, if medical problems were nonsevere, and if the GP was known to the patient beforehand [13].

In this viewpoint, we are therefore primarily concerned with discussing possible barriers to video consultation use among GPs and practice staff, comparing the use and implementation conditions of video consultation between general practice settings in 5 countries (Table 1).

A digital health research network in Denmark, the so-called vCare network, convened an interdisciplinary seminar in May 2022, inviting known experts and existing collaborators from Northern Europe (ie, Denmark, Norway, United Kingdom, the Netherlands, and Germany) who were involved in research on video consultations. The purpose of the seminar was to discuss and debate the differences and similarities in implementation and adoption processes across Northern Europe and to further discuss challenges for future adoption and how research might address this. We were particularly interested in exploring some of the systemic and organizational conditions leading to the current implementation status, illustrating elements that might be crucial for successful adoption in future general practices. To guide our conversations and help generate ideas we had agreed on a set of questions, answers to which might lead to explaining failures and partial successes. We asked the attendees (N=21) from each of the countries to collaborate on collecting information on the following aspects:

- The overall organization and structure of the national health care system and of general practice or primary care
- National and government visions and ambitions for the digitalization of health care
- The trigger of video consultation use and implementation
- Conditions for implementation (ie, technological, sociomaterial equipment and resources, security conditions, financial incentives, and being fit for purpose)
- Volume of use before, during, and after the COVID-19 pandemic
- Challenges: why it has not taken off as expected across our countries and future perspectives

This viewpoint emerges out of that dialogue. After the seminar, the information relating to the above aspects (in written form) was extracted, and quantifiable information obtained from existing sources is presented in Table 1.

The opinions presented in this viewpoint are based on research evidence and clinician experiences. There is, therefore, a risk that biases for or against video consultation influence the viewpoint presented. To increase our reflexivity regarding this point, we had a session at the seminar in which subjective attitudes and the perceived influence of these attitudes on our research were discussed. We also discussed the inherent discourses of the concepts that we use (eg, “implementation,” “adoption,” and “upscaling”) and to which extent our researcher identity within, for example, medical research, implementation research, and social research was shaping the conclusions made. We were careful not to represent “digitalization enthusiasm” or “digitalization criticality.” Representing Northern European health care, our viewpoint can only be transferred to settings with similar conditions and similar digitalization level and readiness.

Discussing the current challenges that general practices face regarding implementation of video consultation and drawing on current literature, we also provide suggestions as to the potential action points that may need to be considered if successful adoption of video consultation is the goal.

**Table 1:** Overview of the implementation conditions of video consultation (VC) by general practitioners (GPs) across countries.

Items	Denmark	Norway	United Kingdom	The Netherlands	Germany
Volume^a^ of VC before COVID-19	0.1%	Less than 3%	0.1%	1% or less	Less than 0.1%
Volume of VC during COVID-19	2.9%	On the highest 5%	0.5% in 2020; 2.6% in 2021	~2% in 2020	~2%
Volume of VC today	1.2%	N/A^b^	2.2%	~3% in 2021	N/A
Mandatory for GPs to provide VC?	Yes, by the end of 2024	No	Yes	No	No
Equipment supply	GPs must arrange for VC through official app free to use for all GPs and patients	GPs are responsible for arranging and paying for one of few VC systems	GPs are responsible for arranging and paying for equipment for one of several VC systems	GPs are responsible for arranging and paying for equipment for one of several VC systems	GPs are responsible for arranging and paying for equipment for one of several VC systems
Video integrated in GPs EPR^c^ system?	No	No; however, some platforms are working on developing an integrated system	Dependent on platform used	Dependent on platform used	Dependent on platform used
GPs financial incentive for VC	Higher reimbursement for VC compared to face-to-face consultations	Same reimbursement for VC as for other types of consultations	No reimbursement for VC; United Kingdom has a capitation system	Same reimbursement for VC as for other types of consultations	GPs may experience less reimbursement for VC than for face-to-face consultations
Patient cost for a VC	Free for patients, same as all GP consultations	Small out-of-pocket fee, same as all GP consultations	None; all care is free at the point of entry	Free for patients, same as all GP consultations, as part of health insurance	Free for patients, same as all GP consultations, as part of health insurance
Health care funding	General taxation	General taxation	General taxation	Social Health Insurance	Social Health Insurance

^a^Apart from the Netherlands, volume of VC indicates the percentage of VC of the total amount of consultations in general practice (including face-to-face, telephone, and e-consultations). In the Netherlands, volume of use was measured through questionnaires as part of the national eHealth monitor. Patients were asked if they had consulted their GP through a VC at least once in the past 12 months.

^b^N/A: not applicable.

^c^EPR: electronic patient report.

## Adoption Reliant on Multilevel Factors

Research on video consultation adoption in general practice has shown that it is reliant on several multilevel factors from health care digitalization level (ie, macro level) to technical infrastructure, IT literacy, and readiness of users (ie, micro level) [6,7,11,14-20]. Across our countries, overlaps were seen in barriers to use relating to technical challenges (eg, poor user-friendliness and low user digital skills), infrastructural (eg, low bandwidth), lacking financial incentives, clinical risks, communicative challenges, no relative advantage to other consultation forms, and lacking integration in the electronic patient record.

Turning to the macro policy level, introducing video consultation as a “need-to” consultation form during COVID-19 only accelerated recent years’ policy-driven ambitions to digitalize general practice in Northern Europe to manage health care demand and related costs, increase efficiency, quality, and access [14].

For example, government policy in the United Kingdom has been focused even before COVID-19 on establishing a “digital-first” primary care [21]. In the Netherlands, the slogan “high-quality care: digital where possible, face-to-face when necessary” [22] shows the recent years’ policy efforts to transition to digital general practice. In Germany, where the digital maturity and infrastructure in health care has developed more slowly, the Medical Association enabled, from 2018, video consultation with patients unable to seek in-clinic contact. In Norway and Denmark, action plans and collective agreements within the organization of general practice seek to promote and stipulate the future use of digital consultations for one-third of all consultations [23].

We argue, however, that there is a misalignment in some countries between policy-level digitalization enthusiasm and the conditions needed for action toward implementation in the clinical context in which GPs and practice staff are operating. This includes ensuring on a national level that technical and financial conditions are met.

## Efforts to Improve the Quality and Supply of Technical Solutions

Across the countries represented here, video consultation is provided by different companies with the common goal of facilitating two-way secure video consultation between GP and patients. Although GPs in Germany and the Netherlands can choose between several solutions with varying user-friendliness, in Denmark, one specific secure solution (the “My Doctor” app) has been endorsed on an organizational level as the “official” solution in general practice and made available for free to GPs and patients. In Norway, GPs must both arrange and pay for the solution themselves creating a further threshold for implementation. In the United Kingdom, there are video consultation options included in the platforms available for providing patients with web-based services in general practice. General practices choose which platform to offer, pay for it, and it is free at the point of use for patients.

## Efforts to Improve Technological Support Are Needed

Currently, not all platforms described in the previous paragraph can be easily integrated into the GP’s electronic patient record system, creating a barrier for using video consultation since the technological setup is then perceived as inefficient. We believe that the integration of the video consulting technological solution in the GPs’ electronic systems is critical for adoption of video consultation in the coming years. This fits with evidence from the United Kingdom showing that integration of technological solutions with GP workflows is a key enabler in increasing GP use [24].

## Efforts to Improve Financial Incentives Are Needed

Focused action aimed at securing sufficient financial support and incentives is needed in several of our countries. Although the implementation of video consultation is supported financially in Denmark and the Netherlands, for example, by agreeing on a reimbursement that is equal or slightly higher than the one for in-person visits (and in Denmark much higher than the reimbursements of both e-consultation and telephone consultation), in Germany, GPs may experience an adverse cost-to-benefit effort due to capped budget. In Norway, billing rates for video consultation are the same as they are for telephone and e-consultations, which does not stimulate GPs to change their mode of contact from telephone to video consultations. In the United Kingdom, there are not individual costs associated with consultation types, with funding attributed at a patient level regardless of consultation type used.

## Focused Action on Understanding Cultural Barriers Is Required

Efforts to improve technological and financial support for the implementation of video consultation need to be accompanied by a focus on understanding, and possibly challenging, GP-perceived barriers toward remote care on a cultural level. By this, we mean the deeply held cultural or professional norms, beliefs, values, and behaviors that relate to the perceived function of general practice and the role of a GP. Evidence shows that in general, GPs are more reluctant than patients about using video consultation [8], due to several perceived barriers, some of them being concerns about patient access to care (equity of care and “the digital divide”) and quality of care within the doctor-patient relationship [18,25].

Consulting the doctor face-to-face has for years been considered the “gold standard” for securing access to care, quality of care, and patient safety. Many GPs and practice staff view and experience the technologically mediated video consultation as constraining their ability to provide high-quality health care to patients. This is due to technological challenges (eg, poor broadband) [26], impaired sensory and semiotic opportunities to read body language (in comparison with face-to-face encounters), and the diminished opportunities that a video consultation provides for being the health professional that they want to be [16,25]. It should thus be recognized that these barriers do not only relate to the individual settings but to wider issues in general practice pertaining to professionalism, identity, and integrity that the medium itself is perceived to challenge.

We argue that if video consultation is ever to become a part of routine general practice, GPs and practice staff need to reflect personally and collectively on how video consultation impacts their professional identity and the norms established in their clinic for how they should work. Research on digital innovation in organizations shows that establishing a shared vision for the adoption process of digital technology is a determinant of success, leading to enhanced commitment, responsibility, and cooperation [27].

In a general practice setting, this process of establishing a shared vision could also involve exploring perceived limitations and potentials of video consultation use for a general practice culture, reflecting for example on attitudes and sense-making toward digital technology, as these have been shown to impact the adoption process of digital technologies to a large extent [28-30]. The perceptions and viewpoints of nonusers should be investigated and taken into consideration in the same way that those of users are considered, and not sidestepped as the presumptions of preusers in need of educational intervention.

We argue that the more we know about GPs’ and general practice staffs’ understanding of and attitudes toward video consultation in the context of their work within general practice, the more we can work on developing educational initiatives that target users’ uncertainties, worries, and “tensions,” hereby reaching a sustainable level of video consultation use that reflect the reality of practice rather than policy optimism and the promissory value of video consultations.

Barriers to use can also be a legitimate reaction to perceptions of inappropriate use or minimal relative advantage for general practice compared to the use of other methods of consulting [6,14]. As GPs have gone back to a primarily face-to-face model post pandemic, continued discussion is needed about whether, when, and for whom video consultation is desirable.

Looking ahead, general practice should consider a new perspective on video consultation beyond viewing it as a crisis and “need-to” tool tied to the COVID-19 pandemic or as “the new normal.” Entering dialogues with other GP colleagues, technology providers, politicians, and patients about their needs and visions for future digital care in general practice seems essential, for example, through cocreative processes and user boards.

Three years following its abrupt and large-scale introduction in general practice, video consultation is still in the process of finding its clear role and advantage—an adaptive process that, as with other health technologies, can be messy, unpredictable, and discontinuous [2,31].

## Recommendations for Future Research

Further research is needed to investigate how to develop, resource, and manage flexible technological and financial solutions required to support appropriate implementation in general practices. This kind of research should be conducted in close collaboration with those invested in implementation processes (eg, health professionals, politicians, government officials, and other stakeholders) to ensure alignment between different interests and perspectives.

Furthermore, research is needed that addresses concerns and skepticisms among users as well as nonusers, including how video consultation practices are perceived to influence professional values and norms, access to care (equity aspects), and quality of care. Addressing issues of safe and ethical use of video consultation, longitudinal studies and register-based research is needed as well as qualitative approaches that enable microanalysis of interaction patterns.

## Conclusions

In this viewpoint, we have explored similarities and differences across our countries regarding the use and implementation conditions of video consultation in general practice within the context of consistently low use of video consultation across the included countries. We have reflected on barriers across general practice settings in our countries, such as lacking technological and financial support for GPs, that we feel are critical for adoption of video consultation in the coming years. Furthermore, there is a need to further investigate the contribution of cultural elements to adoption. This viewpoint may inform policy work to ensure that a sustainable level of video consultation use can be reached in the future, one that reflects the reality of general practice settings rather than policy optimism.

## Abbreviations

GP: general practitioner

## Data Availability

Data sharing is not applicable to this article, as no data sets were generated or analyzed during this study.
